# Lithium Suppresses Astrogliogenesis by Neural Stem and Progenitor Cells by Inhibiting STAT3 Pathway Independently of Glycogen Synthase Kinase 3 Beta

**DOI:** 10.1371/journal.pone.0023341

**Published:** 2011-09-09

**Authors:** Zhenzhong Zhu, Penny Kremer, Iman Tadmori, Yi Ren, Dongming Sun, Xijing He, Wise Young

**Affiliations:** 1 The 2nd Department of Orthopedics Surgery, The 2nd Hospital of Xi'an Jiaotong University, Xi'an City, Shaanxi Province, People's Republic of China; 2 W. M. Keck Center for Collaborative Neuroscience, Rutgers University, Piscataway, New Jersey, United States of America; University of South Florida, United States of America

## Abstract

Transplanted neural stem and progenitor cells (NSCs) produce mostly astrocytes in injured spinal cords. Lithium stimulates neurogenesis by inhibiting GSK3b (glycogen synthetase kinase 3-beta) and increasing WNT/beta catenin. Lithium suppresses astrogliogenesis but the mechanisms were unclear. We cultured NSCs from subventricular zone of neonatal rats and showed that lithium reduced NSC production of astrocytes as well as proliferation of glia restricted progenitor (GRP) cells. Lithium strongly inhibited STAT3 (signal transducer and activator of transcription 3) activation, a messenger system known to promote astrogliogenesis and cancer. Lithium abolished STAT3 activation and astrogliogenesis induced by a STAT3 agonist AICAR (5-aminoimidazole-4-carboxamide 1-beta-D-ribofuranoside), suggesting that lithium suppresses astrogliogenesis by inhibiting STAT3. GSK3β inhibition either by a specific GSK3β inhibitor SB216763 or overexpression of GID5-6 (GSK3β Interaction Domain aa380 to 404) did not suppress astrogliogenesis and GRP proliferation. GSK3β inhibition also did not suppress STAT3 activation. Together, these results indicate that lithium inhibits astrogliogenesis through non-GSK3β-mediated inhibition of STAT. Lithium may increase efficacy of NSC transplants by increasing neurogenesis and reducing astrogliogenesis. Our results also may explain the strong safety record of lithium treatment of manic depression. Millions of people take high-dose (>1 gram/day) lithium carbonate for a lifetime. GSK3b inhibition increases WNT/beta catenin, associated with colon and other cancers. STAT3 inhibition may reduce risk for cancer.

## Introduction

Transplanted neural stem cells (NSCs) produce mostly astrocytes in injured spinal cords, due in part to cytokines released by activated microglia or macrophages [1] e.g. IL-6 [2], [3], ciliary neurotrophic factor [4], [5], or leukemia inhibiting factor [6], [7], NSCs produce relatively few neurons (<20%) that integrate into host spinal cord [8], [9], [10], [11], [12]. When NSC are transplanted as a therapy to replace neurons in injured brain and spinal cord [13], excess astrogliosis may reduce efficacy of the therapies. Astrogliogenesis may also hamper axon outgrowth.

Long used to treat bipolar depression and hematopoietic disorders [14], lithium stimulates NSCs neurogenesis in the hippocampus [15] and subventricular zone [16], causing sustained increases of gray matter volume in patients [17], [18], [19], [20]. Lithium also stimulates transplanted NSCs to produce more neurons [21] as well as axonal growth in injured spinal cord [22], [23]. Other glycogen synthetase kinase (GSK) blockers mimic these lithium effects on neurogenesis and regeneration.

Recent study shows lithium inhibits GSK3β and invokes downstream effects on NSCs development. It increases beta-catenin accumulation [24], which combines with WNT to stimulate NSC proliferation and neurogenesis. RNAi inhibition of beta-catenin abolishes these lithium-induced effects [25]. Beside the effect on stimulating NSCs proliferation and neurogenesis, lithium is also found reducing astrogliogenesis by NSCs [26], but the mechanisms underlay remains an enigma.

Lithium inhibits multiple messenger systems [27], [28], including the JAK/STAT3 pathway [29] known to stimulate astrocytosis [30]. We therefore studied the effects of lithium and other GSK3β blockers on astrogliogenesis by NSCs isolated from neonatal rat brains. Both lithium and another GSK3β inhibitor SB216763 stimulated neurogenesis but only lithium suppressed astrogliogenesis by NSCs. In addition, analysis of restricted progenitor cell proliferation revealed that both lithium and SB216763 promotes neuronal restricted progenitor (NRP) cell proliferation, but only lithium inhibited the proliferation of GRPs. Further investigation showed that lithium not only strongly inhibited STAT3 activation, but also abolished the effect of a STAT3 agonist AICAR on inducing STAT3 activation and astrogliogenesis, indicating that lithium suppresses astrogliogenesis through inhibiting STAT3. Nevertheless, neither specific GSK3β inhibitor SB216763 nor molecular blockade of GSK3β with GID5-6 overexpression inhibited astrogliogenesis or STAT3 activation induced by serum or AICAR, These results together indicate that lithium inhibits astrogliogenesis through a non-GSK3β-mediated inhibition of STAT3.

## Results

### Neural Stem Cells and Progenitor Cells

**Figure 1:**
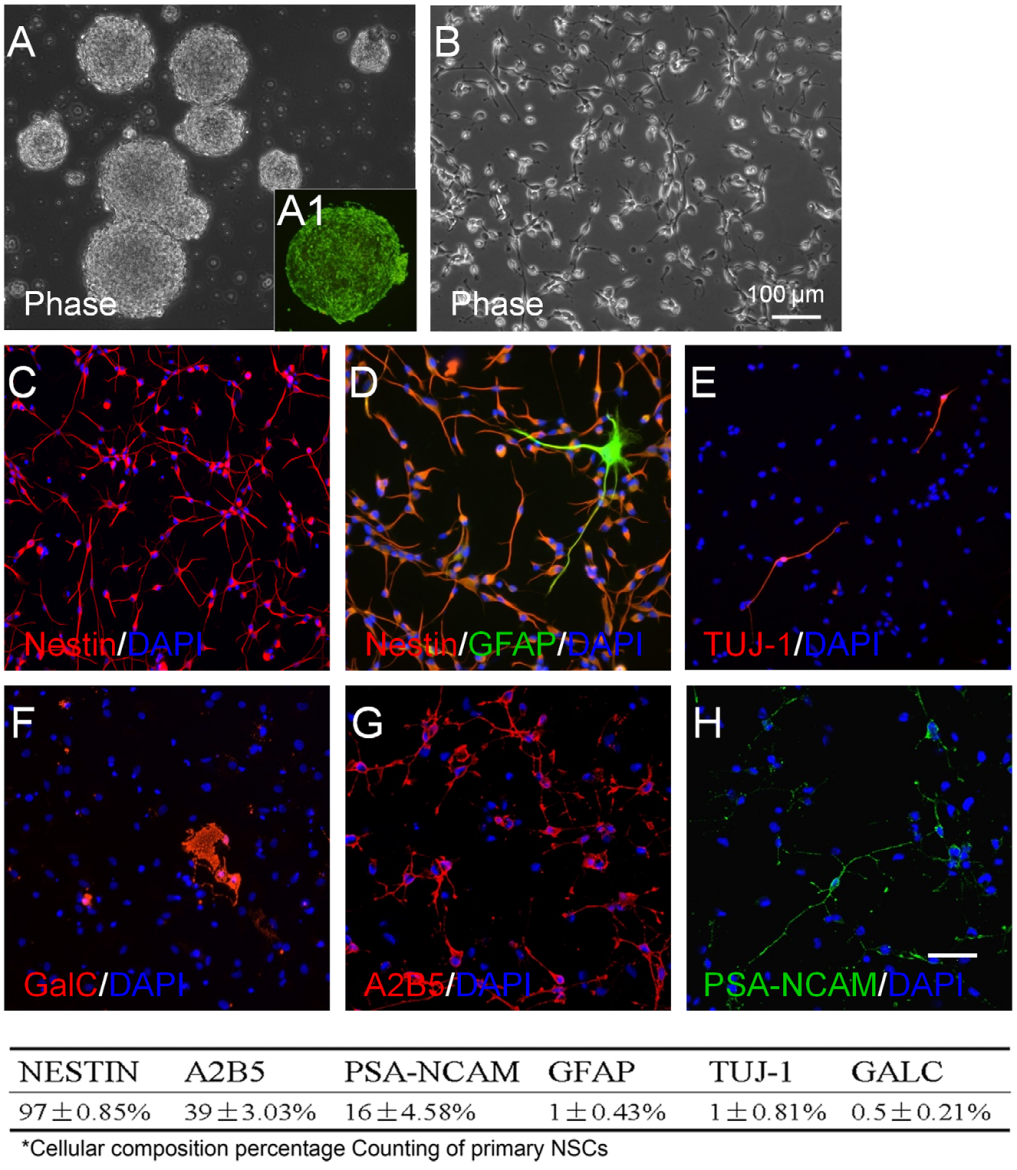
Heterogeneity of primary NSCs. Primary rat NSCs were cultured in growth medium containing 10 ng/ml bFGF and 10 ng/ml EGF for 7 days. **A.** Neurospheres formed and expressed nestin (A1, green). **B.** A phase image of adherent NSCs. **C–H.** Immunostaining of nestin (C, red), GFAP (D, green), Tuj1 (E, red), and GalC (F, red), A2B5 (G, red) and PSA-NCAM (H, green). Nuclei were stained with Hoechst 33342 (DAPI, blue). The table lists percentages of each cell subpopulation relative to total cell count. Data are expressed as mean ± sem averaged from four independent experiments. The scale bar indicates 100 µm.

Growing NSC in growth media expectedly produced heterogeneous cultures of cells that expressed neuronal, astrocytic, and oligodendroglial markers. After 7 days in growth in serum-free media (DMEM with bFGF and EGF), NSCs proliferated and congregated in loose colonies that expressed nestin (Figure 1A), an intermediate filament protein present in NSC and progenitor cells [37]. The cells often formed neurospheres. After dissociation, replating, and growth in 10 ng/ml bFGF and EGF for 24 hours, the cells were unipolar or bipolar with short processes (Figure 1B) and almost all (97±0.85%) expressed nestin (Figure 1C). Very few cells (1.00±0.43%) expressed GFAP, characteristic of mature astrocytes (Figure. 1D). Likewise, only 1.00±0.81% expressed Tuj1 (Figure. 1E), a neuronal marker. Only 0.50±0.21% expressed GalC (Figure 1F), a major myelin galactosphingolipid and oligodendroglial marker. Many cells expressed A2B5 or PSA-NCAM, presumptive markers [38], [39] for GRPs and neuronal restricted progenitors (NRPs) respectively comprising 39.0±3.03% (Figure 1G) and 16.0±4.58% (Figure 1H) of the cultures.

**Figure 2:**
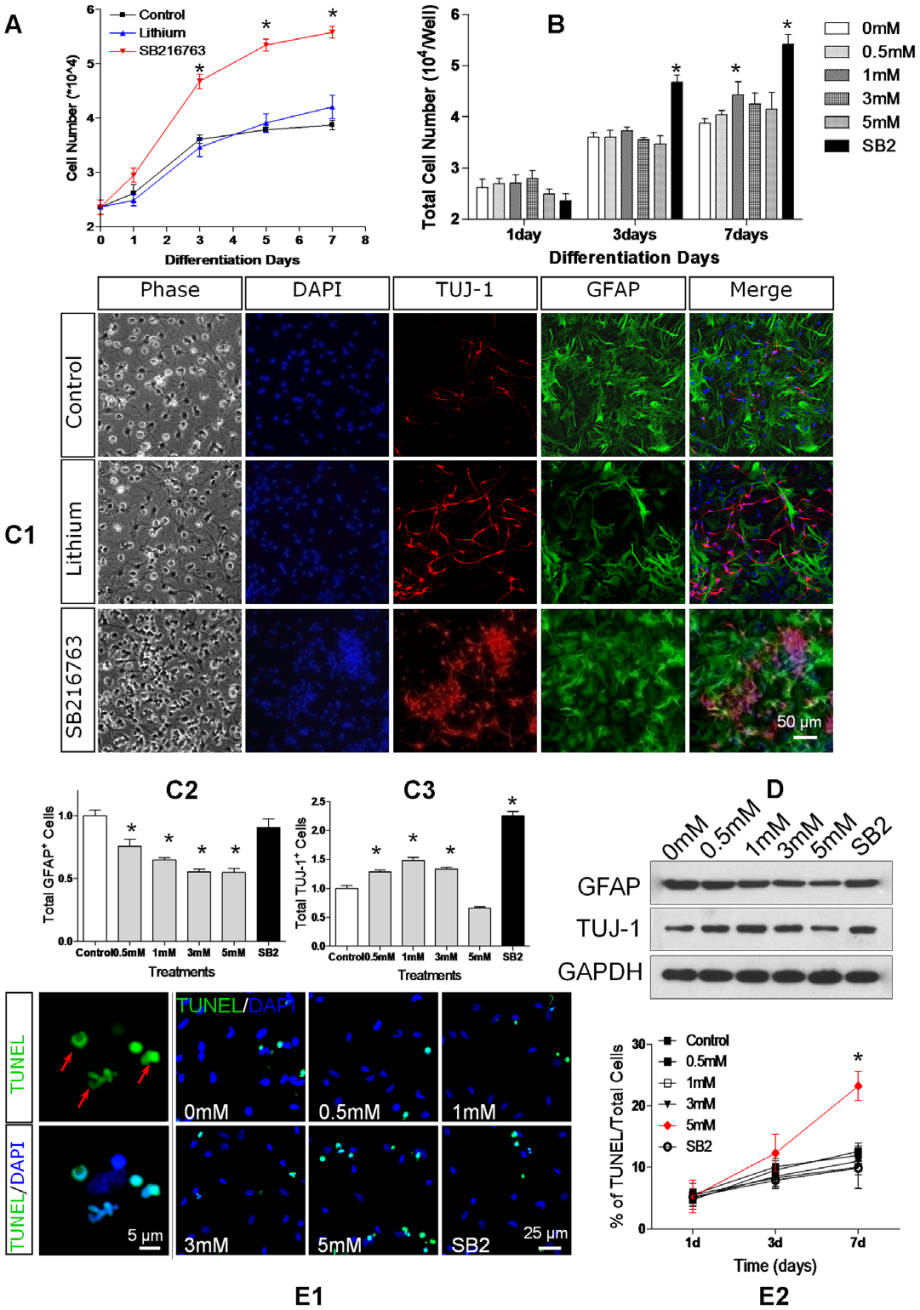
Inhibition of GSK3β regulates NSC differentiation. **A.** Rat NSCs were cultured in NB27 medium supplemented with LiCl (1 mM), SB216763 (10 µM), or no treatment (control). Cell numbers were estimated with the CyQUANT assay. **B.** The total cell numbers of untreated (0 mM), LiCl-treated (0.5, 1.0, 3.0, 5.0 mM) and SB216763-treated (10 µM) cultures at 1, 3 and 7 days after passage. **C.** NSCs were grown for 7 days in NB27 with LiCl (0.5, 1.0, 3.0, 5.0 mM) or SB216763 (10 µM) and then stained for DAPI (blue), Tuj1 (red), and GFAP (green). The photomicrographs (C1) show representative fields from each treatment group (3 mM Lithium, scale bar = 50 µm). The graphs show actual number counts of GFAP+ cells (C2) and Tuj1+ cells (C3), normalized to untreated control counts. **D.** The expression of GFAP and Tuj1 on NSCs treated with LiCl (0, 0.5, 1.0, 3.0, 5.0 mM) or SB216763 (SB2, 10 µM) after growing 7 days. **E1.** NSCs were treated with LiCl (0, 0.5, 1.0, 3.0, 5.0 mM) or SB216763 (SB2, 10 µM) for 7 days and apoptotic cells were detected by TUNEL assay (E1, green). Red arrows indicate the typical morphology of apoptotic cells (left two panels, scale bar = 5 µm), the right 6 panels show the staining of TUNEL+ cells in different treatment groups. Nuclei were stained with Hoechst 33342 (blue, scale bar = 25 µm). **E2.** Percentages of TUNEL+ cells treated with LiCl and SB216763 at indicated time. Data are expressed as mean ± sem from three independent experiments (n = 3, * denotes P<0.05 vs. control, one way ANOVA with Dunnett's post-test).

When transferred to neurobasal media containing B27 (NB27, Invitrogen), many of the cells began to show mature neuronal, astrocytic, and oligodendroglial markers. After 7 days in NB27 media, 32.1±1.4% of the cells expressed the neuronal marker Tuj1 and 42.8±1.9% expressed the astrocytic marker GFAP (Figure 2C1). The cells expressing Tuj1 or GFAP also had respectively the morphology of neurons and astrocytes. In general, astrocytes outnumbered neurons.

In summary, NSC cultures produced progenitor and differentiated cells. In serum-free growth media, the cells proliferated and formed loose colonies of nestin-expressing cells and neurospheres. Many cells expressed A2B5 or PSA-NCAM, presumptive markers for glial-restricted and neural-restricted progenitor cells. Relatively few cells expressed mature neuronal, astrocytic, and oligodendroglial markers. However, when grown for 7 days in NB27 medium, NSCs produce mostly GFAP+ astrocytes and Tuj1+ neurons, the former more than the latter.

### GSK3β Inhibition Promotes NSCs Proliferation in NB27 Medium

Lithium and other GSK3β inhibitors stimulate growth of NSC and progenitor cells [21], [25], [40]. We therefore examined the effects of lithium and the GSK3β inhibitor SB216763 on NPC's grown in NB27 medium. Even in the absence of added growth factors, NSCs and NPC's continued to proliferate in NB27 (Figure 2A) and SB216763 was more potent than lithium in stimulating proliferation in NB27 without growth factors. Lithium (1 mM) increased total cell number by 1.1 fold after 7 days. In contrast, SB216763 (10 µM) markedly stimulated NSC proliferation, increasing total cell number by 1.4 fold (p<0.05 compared to lithium-treated cultures) after 7 days (Figure 2B).

### Lithium but not SB216763 Suppresses Astrogliogenesis

After 7 days in NB27, many cells began showing mature astrocytic marker GFAP and neuronal marker Tuj1. We stained and counted the cells, estimating the total numbers and percentage of cells of each lineage. While both lithium and SB216763 enhanced neurogenesis, only lithium suppressed astrogliogenesis. Lithium significantly increased the number of Tuj1 positive cells by 1.28±0.03 fold in 0.5 mM LiCl (P<0.05), 1.47±0.06 fold in 1.0 mM LiCl (P<0.05), 1.33±0.03 fold in 3.0 mM LiCl (P<0.05), 0.66±0.02 fold in 5.0 mM LiCl (P<0.05), 2.25±0.07 fold in 10 µM SB216763 (P<0.05) from control level (Figure 2C3). Conversely, lithium reduced the GFAP-positive cells number from control level to 0.75±0.05 fold in 0.5 mM LiCl (P<0.05), 0.64±0.02 in 1.0 mM LiCl (P<0.05), 0.55±0.02 in 3.0 mM LiCl (P<0.05), 0.54±0.03 in 5.0 mM LiCl (P<0.05). Conversely, SB216763 treatment did not reduced astrocytes number (0.90±0.06 fold versus Control, P>0.05, Figure 2C2). We also found the similar tendency with S100beta immunostaining (Figure S1), which is another astrocytes marker. These results indicate that lithium and SB216763 exert different effects on astrogliogenesis.

Western blots confirmed the decrease of GFAP and increase of Tuj1 protein in the lithium-treated cultures (Figure 2D). Compared to control untreated cultures, 5 mM LiCl treatment reduced GFAP expression by 50% (p<0.01) and LiCl treatment increased Tuj1 expression, particularly at 1 mM. Lithium had a higher dose-response curve for suppressing astrogliogenesis than neurogenesis. The inhibitory effects of lithium on astrocytic production continued to increase up to 5 mM while the stimulatory effects of lithium on neurons peaked at 1 mM.

Lithium suppression of astrogliogenesis was most prominent at lithium concentrations ≥3 mM. At these doses, lithium may be toxic to cells, hence reducing the number of astrocytes by causing apoptosis [41] rather than by inhibiting astrogliogenesis. We therefore studied the effects of lithium and SB216763 on apoptosis in the cultures, using the TUNEL assay [42], [43]. Neither 10 µM SB216763 nor 1–3 mM LiCl increased apoptosis of cells cultured in NB27 medium for 7 days. However, higher (5 mM) concentrations of LiCl nearly doubled the number of TUNEL-positive cells (Figure 2E). While lithium toxicity may partly explain the decline in astrocytes at 5 mM, it cannot account for the reduction of astrocyte count at 3 mM. we observed that lithium and SB2 induced neurite spreading and branching, which may duo to their inhibition on GSK3β [44].

In summary, analysis of cell counts of Tuj1- and GFAP- expressing cells revealed that, while both lithium and SB216763 stimulated neurogenesis, only lithium suppressed astrogliogenesis. SB216763 increased the number of neurons but did not reduce the number of astrocytes. Lithium increased the number of neurons and reduced the number of astrocytes. Higher concentrations of lithium (3 mM) were required to suppress astrogliogenesis than to stimulate neurogenesis (1 mM). Although lithium increased apoptosis of astrocytes at 5 mM, it did not do so at 3 mM, suggesting that the reduction of astrocytes at 3 mM was not due to lithium-induced apoptosis.

### Lithium and SB216763 Effects on GRP Proliferation

**Figure 3:**
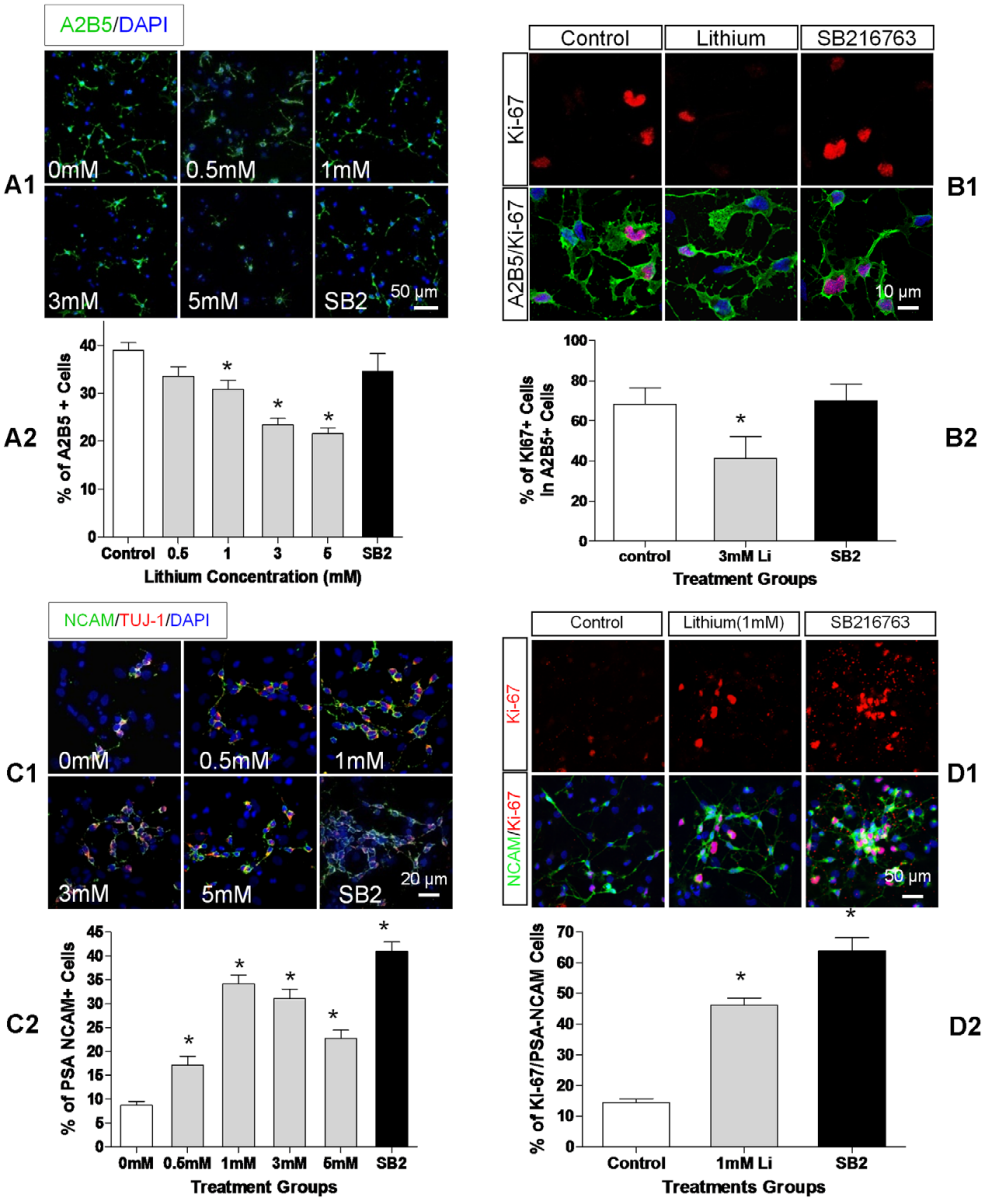
Inhibition of GSK3β differentially regulates progenitor cell proliferation. **A1.** Immunostaining for A2B5 (green) and Hoechst 33342 (blue). NSCs were grown for 2 days in NB27 media containing LiCl (0, 0.5, 1.0, 3.0, 5.0 mM), or SB216763 (SB2, 10 µM). **A2.** Quantification of A2B5+ cells as a percentage of total cell count for each LiCl dose and SB216763. **B1.** The proliferating A2B5+ cells identified by immunostaining for Ki-67 (red) and A2B5 (green). Cells were treated with LiCl or SB216763 for 24 h. **B2.** Percentages of Ki-67+ cells amongst A2B5+ cells cultured for 24 h in control, 3 mM LiCl, or SB216763-treated NSCs. **C1.** PSA-NCAM+ (green) co-localized with Tuj1 (red) at this stage, pictures show the percentage of PSA-NCAM+ cells in NSCs treated with LiCl (0, 0.5, 1, 3, 5 mM) or SB216763 (10 µM) for 5 days. **C2.** The percentage of PSA-NCAM+ cells relative to total cell count. **D1.** The proliferating PSA-NCAM+ cells identified by immunostaining with Ki-67 (red) and PSA-NCAM (green) after 4 days. **D2.** The percentage of double Ki-67+/PSA-NCAM+/cells among total PSA-NCAM+ cells. Data are expressed as mean ± sem from three independent experiments, * denotes P<0.05 vs. control (one way ANOVA with Dunnett's post-test).

Both lithium and SB216763 reduced the percentage of GRPs, as measured by A2B5 immunostaining. The percentage of A2B5+ cells fell from 39±1.6% in control cultures to 25±1.7% after 2 day in 3 mM LiCl culture. SB216763 also reduced the percent of A2B5+ cells but not as much as lithium (Figure 3A). Combining the percentage and cell count data (Figure 2A) indicated that the actual number of GRPs increased with time in untreated and SB216763-treated cultures. Lithium-treated cultures however, showed little or no increase in A2B5+ cell counts, suggesting that lithium inhibits proliferation or production of A2B5+ cells.

To confirm this hypothesis, we stained the cells for Ki-67, a nuclear and nucleolar protein that increases with somatic cell proliferation [45], [46]. Lithium dramatically reduced the Ki-67 positive fraction of A2B5+ cells to 38%, compared to 68% in control untreated cultures. In SB216763-treated cultures, Ki-67 positive fraction was 64%, not significantly different (p>0.05) from control untreated cultures (Figure 3B).

In summary, lithium (3 mM) reduced both the percentage and the number of GRPs but GSK3β blocker SB216763 did not. Lithium markedly reduced the fraction of A2B5+ cells that stained for Ki-67, a nuclear marker that reflects cell division, from 68% in untreated control cultures to 38%. SB216763, however did not significantly alter the fraction of Ki-67 labeled A2B5+ cells, confirming that lithium but not SB216763 suppressed proliferation of GRPs.

### Lithium and SB216763 Effects on NRP Proliferation

To assess whether lithium and SB216763 stimulated proliferation of NRP cells, we assessed the effects of these drugs on PSA-NCAM expressing cells. Most Tuj1+ cells co-localized with PSA/NCAM after 5 days of differentiation (Figure S3). Both lithium and SB216763 significantly increased percentages of PSA-NCAM+ cells in 5-day NB27 cultures, i.e. 9% in control cultures compared to 34% in 1 mM LiCl and 41% in 10 µM SB216763-treated cultures (Figure 3C). Analysis of the cell counts indicates that both drugs significantly increased the number of NRPs in the culture.

Double staining for Ki-67 and PSA-NCAM revealed that both lithium and SB216763 robustly increased the Ki-67 fraction of PSA-NCAM+ cells, i.e. from 14% in control cultures to 51% in 1 mM LiCl and 64% in SB216763-treated cultures (Figure 3D). This suggests that both lithium and SB216763 enhanced production or proliferation of NRPs in the cultures.

In summary, GSK3β blockade by lithium or SB216763 stimulated production of more neurons. Double staining for Ki-67 and PSA-NCAM revealed that both drugs enhanced production or proliferation of NRPs.

### Lithium Effects on JAK/STAT3 Activation and Astrogliogenesis

JAK/STAT3 regulates astrocytic production by NSCs. Molecular suppression of STAT3 gene expression or pharmacological inhibition of STAT3 activity markedly reduces astrogliogenesis [30], [47], [48]. One recent study [29] reported that very high concentrations of LiCl (20 mM) blocked STAT3 activation induced by lipopolysaccharide (LPS) or interferon in astrocytes. Since our experiments showed that lower LiCl concentrations (3 mM) suppressed astrocytosis, we were interested to know whether this concentration of lithium would block STAT3 activation induced by gentler stimuli.

**Figure 4:**
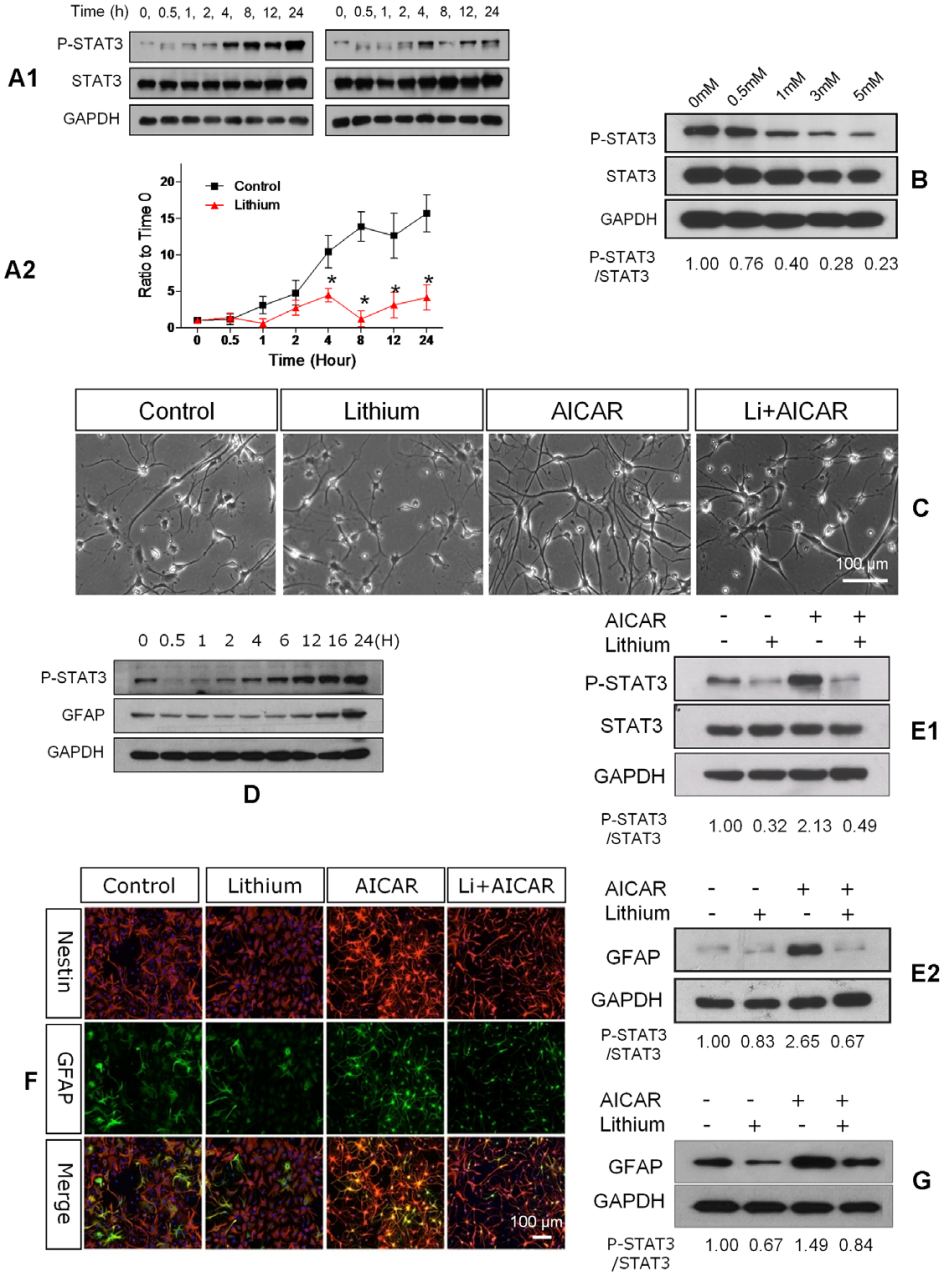
Lithium suppresses STAT3 activation. **A1.** LiCl inhibits serum-induced STAT3 activity in a time-dependent manner. Primary rat NSCs were cultured in NB27 medium with 0.5% of FBS in the absence (left) or presence of 3 mM lithium (right) for the indicated time. The STAT3 activity was assessed by detection of phospho-Tyr705-STAT3 (p-STAT3). Similar results were obtained from three independent experiments. **B.** LiCl inhibits serum-induced STAT3 activity in a dose-dependent manner. NSCs were cultured with various concentrations of LiCl (0.5, 1, 3, 5 mM) in the presence of serum for 24 h. **C.** Morphological changes of NSCs treated with LiCl, AICAR and LiCl+AICAR, respectively. NSCs received no treatment (Control), lithium (3 mM), AICAR (1 mM), or 45 minutes of lithium pretreatment and addition of AICAR (Li+AICAR). Phase contrast images indicate typical astroglia morphology. **D.** AICAR induced STAT3 activation and GFAP expression in a time dependent manner. NSCs were treated with 1 mM AICAR for the indicated time and P-STAT3, GFAP and GAPDH were assessed by Western Blot analysis. **E.** STAT3 (E1) activation and GFAP (E2) expression on NSCs treated with AICAR, lithium or both for 24 hours. **F.** Expression of Nestin (red) and GFAP (green) on NSCs treated with AICAR, lithium or both for 3 days. **G.** GFAP expression on NSCs treated with AICAR, lithium or both for 3 days.

Serum induces astrogliogenesis through STAT3 activation [49]. We grew NSCs in NB27 medium for 7 days with 0.5% serum and used Western blots to measure phosphorylated Tyr705 STAT3 (P-Tyr705-STAT3). Phosphorylation at Tyr-705 activates STAT3, causing dimmer formation, nuclear translocation, and regulation of gene expression [50]. P-Tyr-705 STAT3 started increasing by 2 hours and reached 17× baseline (time 0) levels at 24 hours. Application of 3 mM lithium suppressed P-Tyr705-STAT3 to 4× baseline at 24 hours (P<0.01, Figure 4A). Lithium reduced expression of P-Tyr705-STAT3 in a dose dependent manner after 24 hour of treatment (Figure 4B). Total STAT3 did not change at all time points.

The STAT3 agonist AICAR induces astrogliogenesis by activating STAT3 [51]. Applying 1 mM AICAR markedly increased cells exhibiting astrocytic morphology (Figure 4C), stimulated P-Tyr705-STAT3 and GFAP expression (Figure 4D) after 24 hours. However, treatment of 3 mM LiCl dramatically reduced the cells of typical glia morphology (Figure 4C) and suppressed the induced up-regulation of P-Tyr705-STAT3 and GFAP (Figure 4E) after 24 h. Immunostaining showed that lithium markedly reduced GFAP expression after 3 days in both control untreated and AICAR-treated cultures (Figure 4F, 4G).

In summary, both serum and AICAR stimulate astrocytosis by activating STAT3. We confirmed that adding 0.5% serum increased P-Tyr705-STAT3 to 17× of baseline (pre-treatment) levels, associated with increased astrocytosis and GFAP at 24 hours. Adding 3 mM LiCl reduced P-Tyr705-STAT3 to 4× of baseline and prevented the astrocytosis. The STAT3 agonist AICAR likewise activated STAT3 and increased astrocytes and expression of P-Tyr705-STAT3. Applying 3 mM LiCl to the culture dramatically reduced the number of cells expressing GFAP in control and AICAR-treated cultures. These data indicate that lithium blocks STAT3 activation and prevents astrocytosis.

### Effect of SB216763 on JAK/STAT3 Activation and Astrogliogenesis

**Figure 5:**
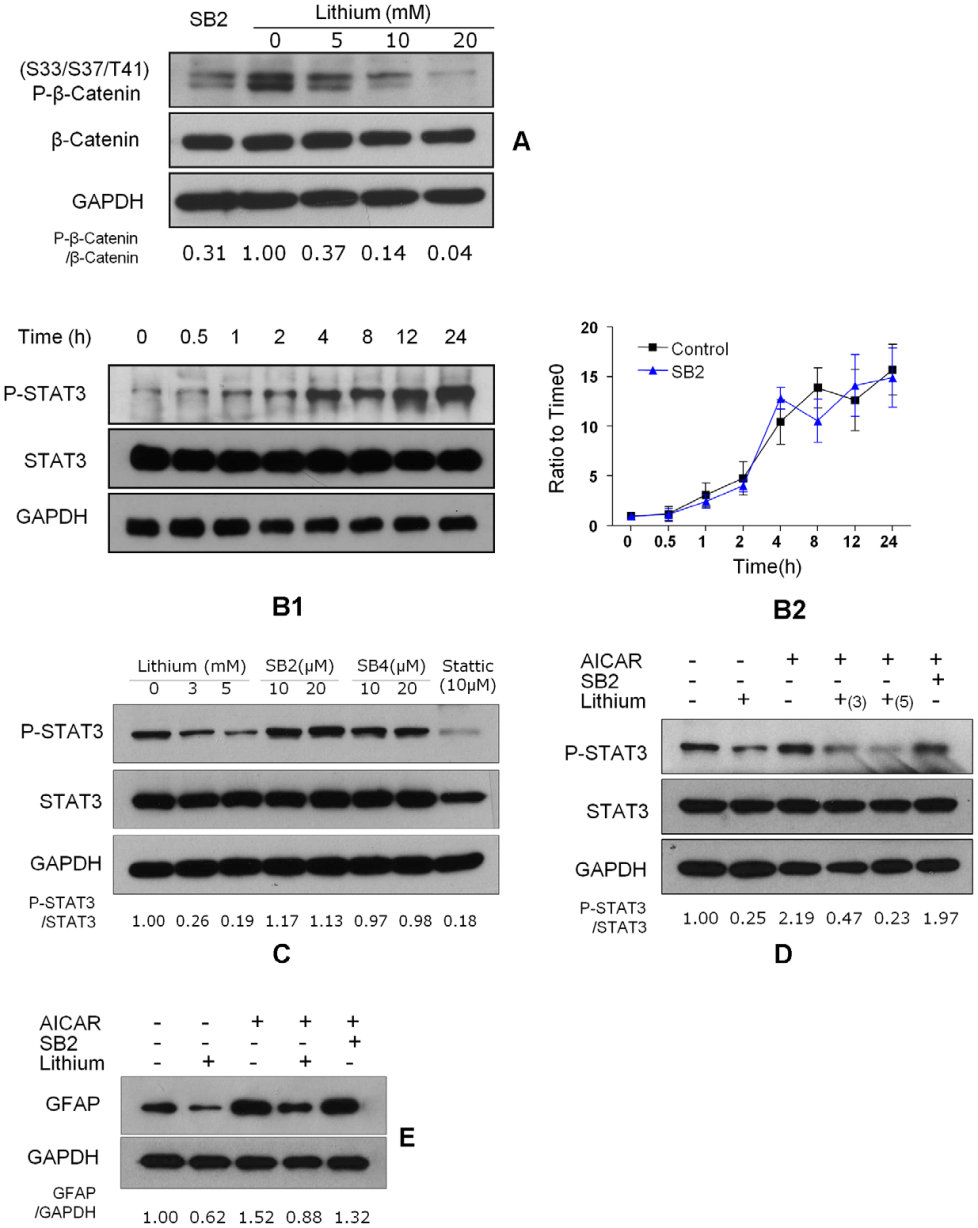
Specific GSK3β blockade has no effect on STAT3 activation and astrogliogenesis. **A.** Lithium and GSK3β blocker SB216763 inhibit beta catenin phosphorylation (p-beta-Catenin). NSCs were treated with SB216763 (SB2, 10 µM) and LiCl (0, 5, 10, 20 mM) for 30 min. The GSK3β activity was assessed by detection of p-beta-Catenin. **B1.** SB216763 had no effect on serum-induced STAT3 activation. NSCs were cultured in NB27 medium with 0.5% FBS in the presence of 10 µM SB216763 for the indicated time. **B2.** Serum increased p-STAT3 over time and SB216763 did not change this curve. Data are expressed as mean ± sem, averaged from three independent experiments and normalized to control values (n = 3, * P<0.05 vs. control, # P<0.05 vs. SB2 treatment group, one way ANOVA with Dunnett's post-test). **C.** STAT3 activation on NSCs treated with lithium and specific GSK3β inhibitors SB216763 and SB415286. NSCs were treated with LiCl, SB216763, SB415286 and STAT3 inhibitor Stattic at indicated concentrations for 24 h. **D.** STAT3 activation on NSCs incubated with 1 mM AICAR for 24 h with or without a 45-minute pretreatment of LiCl (3/5 mM) or SB216763 (SB2, 10 µM). **E.** GFAP expression on NSCs stimulated with AICAR for 3 days in the presence or absence of lithium.

Before assessing the effects of the GSK3β blocker SB216763 on STAT3 activation and astrocytosis, we verified that lithium and SB216763 blocked GSK3β mediated phosphorylation of beta-catenin, a widely used assay of GSK3β activity [34], [36], [52]. As shown in Figure 5A, 30 minutes of treatment with 5–20 mM of LiCl significantly reduced phosphorylated beta-catenin (p-beta-catenin). SB216763 likewise inhibited formation of p-beta-catenin.

Exposing NSC cultures to 0.5% serum significantly increased activated STAT3 (P-Tyr-705-STAT3) by 2 hours and to very high levels by 24 hours (Figure 5B). Both 3 and 5 mM LiCl reduced P-Tyr705-STAT3 levels compared to 0 mM LiCl but neither 10 nor 20 µM SB216763 reduced P-Tyr705-STAT3 levels (Figure 5C). Another GSK3β blocker SB415286 did not reduce P-Tyr705-STAT3 either. In fact, both drugs increased P-Tyr705-STAT3 levels slightly. The Jak/STAT3 inhibitor Stattic (10 µM) markedly reduced P-Tyr705-STAT3 levels. Similarly, 3–5 mM lithium reduced P-Tyr705-STAT3 induced by the STAT3 agonist AICAR but SB216763 did not (Figure 5D). Lithium reduced GFAP levels, AICAR increased GFAP, lithium partially blocked the AICAR induced GFAP rise, but SB216763 did not (Figure 5E).

In summary, pharmacological blockade by SB216763 did not block STAT3 activation, manifested by no differences of P-Tyr705-STAT3 levels induced by serum or AICAR. We confirmed that SB216763 blocked GSK3β mediated phosphorylation of beta-catenin and is approximately 1000 times more potent than lithium. Increasing the dose of SB216763 to 20 µM did not block STAT3 either. Another GSK3β blocker SB415286 did not prevent the STAT3 activation by serum. SB216763 also did not block AICAR-induced increase in GFAP. In contrast, lithium blocked the AICAR-induced rise in P-Tyr705-STAT3 and reduction of GFAP.

### Effect of GID5-6 on STAT3 activation and astrogliogenesis

**Figure 6:**
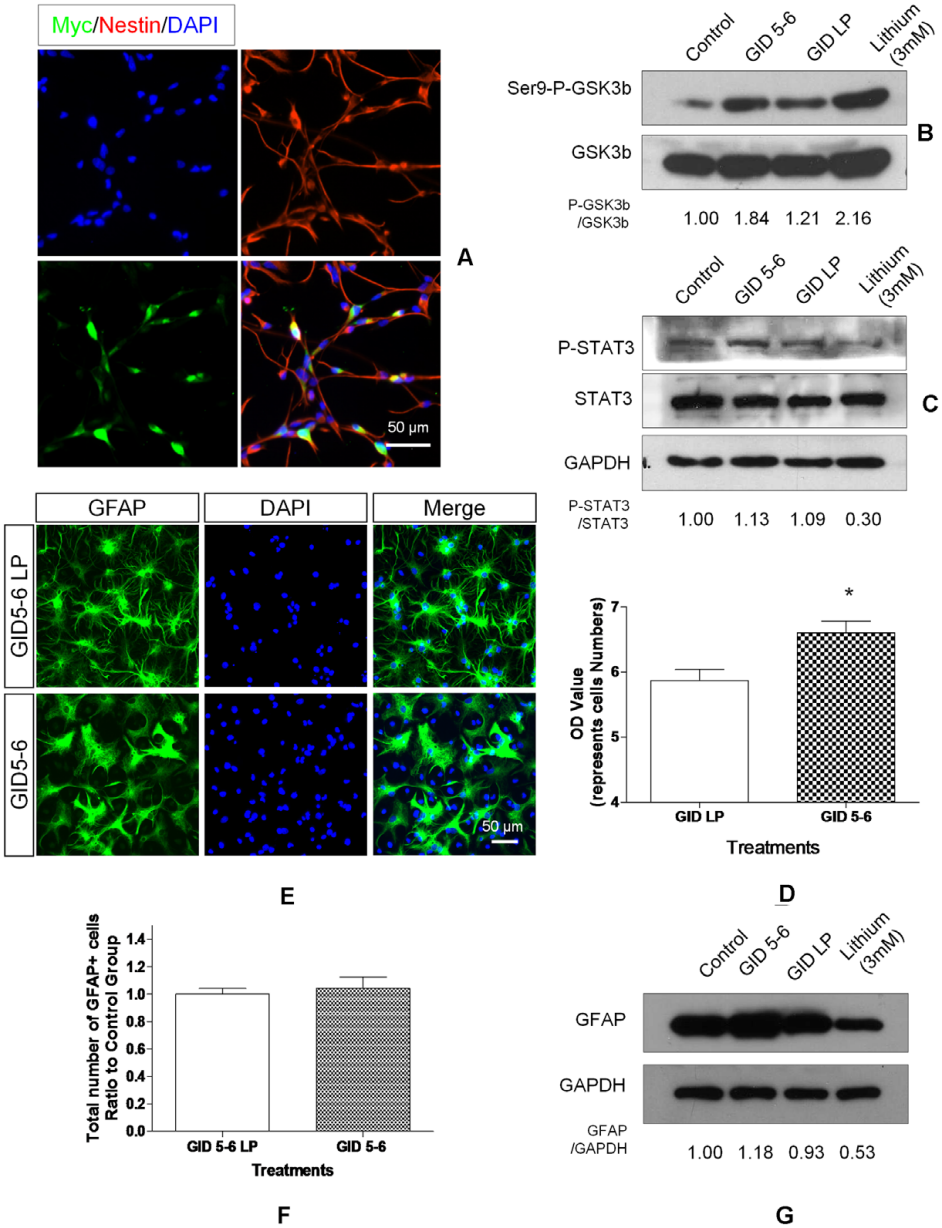
GSK3β inhibition by GID 5-6 does not mimic lithium effect. NSCs were transfected by electrophoresis with liposomes containing DNA to make Myc-labelled GID5-6, which binds GSK3β and prevents its docking to the cytoplasmic protein axin and phosphorylating beta-catenin. GID5-6/LP is an ineffective analog of GID5-6. **A.** Transfection efficiency was assessed by immunostaining the cells for Myc (green) after 24 h. Most of the cells were nestin+ (red). **B.** The effect of GID 5-6 transfection on GSK3β activity. GSK3β activity was assessed by immunoblotting for GSK3β phosphorylated at Ser9 (Ser-P-GSK3β). **C.** The effect of GID 5-6 transfection on STAT3 activation on NSCs incubated with 0.5% FBS for 24 hours. **D.** GID5-6 transfection increased cell numbers by 1.2 fold versus GID5-6 LP transfection group as measured by CyQUANT Assay. **E.** Neither GID5-6 nor GID5-6 LP affected the number of GFAP expressing cells. **F.** GID5-6 transfection had no effect on number of GFAP-expressing cells. Data were expressed as mean ± sem obtained from three independent experiments (n = 3, * p<0.05 vs. control, t -test). **G.** GID5-6 transfection did not affect GFAP level on NSCs but lithium (3 mM) markedly reduced GFAP level.

GID5-6 is a specific molecular blocker of GSK3β, overexpression of GID5-6 inhibits GSK3β activity in vitro. The GID5-6 and GID5-6LP were myc-tagged so that we could tell which cells were transfected. The Amaxa® Nucleofector® Kit yielded 50–60% transection efficiency (Figure 6A). Transfection with GID5-6 upregulated GSK3β phosphorylation, identified with a Ser-9 GSK3β antibody and indicative of GSK3β inhibition (Figure 6B). However, neither GID5-6 nor GID5-6LP blocked the increase of P-Tyr705-STAT3 induced by 0.5% serum while lithium did (Figure 6C). GID5-6 transfection increased total cell numbers after seven days (1.2×, n = 3, p<0.05) compared to GID5-6LP transfection (Figure 6D, 6E) but not the number of GFAP-expressing cells (Figure 6F, 6G).

In summary, transfection and overexpression of GID5-6 effectively inhibited GSK3β activity and stimulated proliferation of NPC but did not stop inhibition STAT3 phosphorylation or GFAP production. Thus, lithium inhibits STAT3 activation and astrogliogenesis through a mechanism not involving GSK3β.

## Discussion

Wexler, et al. [25] previously reported that lithium stimulates hippocampal neurogenesis by inhibiting GSK3β and elevating beta-catenin. Our experiments confirmed that both lithium and the GSK3β blocker SB216763 stimulated neurogenesis in NSC cultures grown in NB27 medium, increasing both the proportion and number of cells that express PSA-NCAM, as well as the production of Tuj1, as determined by Tuj1 single and BrdU/Tuj1 double staining (Figure S2). Lithium also reduced the proportion and number of cells expressing A2B5, as well as cells expressing the mature glial marker GFAP.

Several investigators have noted these inhibitory effects of lithium on glial cells [26], [53], our further investigation showed that lithium prevented increases in the number of A2B5+ and GFAP+ cells in NSC cultures but SB216763 did not. In lithium-treated cultures, counts of A2B5+ and GFAP+ cells did not increase as much as in untreated cultures. In SB216763-treated cultures, the number of A2B5+ and GFAP+ cells increased and did not differ from untreated cultures. This is the first evidence suggesting that lithium suppressed astrogliogenesis may not through non-GSK mechanisms.

We hypothesized that lithium blocks phosphorylation of STAT3, a messenger system known to stimulate astrogliogenesis. To test this hypothesis, we measured P-Tyr705-STAT3 as an indicator of STAT3 activation. Adding 0.5% serum or the specific STAT3 agonist AICAR rapidly increased P-Tyr705-STAT3 protein and GFAP levels in NSC cultures. Lithium blocked this P-Tyr705-STAT3 and GFAP increase with the same dose-response as it inhibited astrogliogenesis. Neither SB216763 nor GID5-6, a highly specific molecular blocker of GSK3β blocked induced P-Tyr705-STAT3 or GFAP increases. Together these results provide convincing evidence that lithium inhibits astrogliogenesis in NSC cultures by preventing STAT3 phosphorylation through non-GSK3β mechanisms.

In contrast, GSK3β inhibition stimulates neural progenitor cells to proliferate. Both lithium and SB216763 markedly increased the fraction of Ki-67+ cells amongst PSA-NCAM+ cells but not A2B5+ cells. Ki-67 is a marker of nucleolar and nuclear proteins expressed by dividing or recently divided cells. In control untreated cultures, only 14% of PSA-NCAM+ cells labeled for Ki-67 compared to 51% in 1 mM lithium-treated cultures and 64% in 10 µM SB216763-treated cultures.

Lithium clearly inhibits STAT3 in NSC cultures. Beurel & Jope [29], [54], [55] had earlier reported that STAT3 activation depends on GSK3β in astrocytes and microglia. They found that 20 mM lithium and other drugs that blocked GSK3β and suppressed STAT3 activation induced by lipopolysaccharide (LPS) and interferon-gamma in mouse primary astrocytes and microglia. Like Beurel & Jope, we found that lithium inhibits STAT3. However, unlike Beurel and Jope, we found that SB216763 did not block serum- or AICAR-activation of STAT3. We thus chose to test another and more specific GSK3β blocker, i.e. GID5-6, to see if it would inhibit serum- or AICAR activation of STAT3. We speculate this discrepancy might be due to the different culture condition and the dominance of regulating pathways among different cell types.

The cytoplasmic protein axin plays a critical role in GSK function [36]. In order for GSK3β to phosphorylate (inactivate) beta-catenin, both molecules must bind to axin. GID5-6 is the part of axin that specifically binds GSK3β. While overexpression of full length axin will cause more inactivation of beta-catenin, expression of GID5-6 should inhibit GSK3β and prevent beta-catenin phosphorylation. We confirmed that expression of GID 5-6 blocked GSK3β activity and phosphorylation of beta catenin in NSCs. However, GID 5-6 did not affect serum- or AICAR-induced STAT3 activation or astrogliogenesis. These results indicate that specific blockade of GSK3β does not prevent STAT3 activation by serum or AICAR.

Thus, our data indicate that GSK3β blockade does not necessarily inhibit STAT3 activation in NSC cultures. While GSK3β may play an important role activating STAT3 in astrocytes and microglia stimulated by LPS and interferon gamma [29], [54], [55], GSK3β does not seem to do so in NSC cultures stimulated by gentler STAT3 agonists. The effect of lithium on STAT3 and astrogliogenesis appears to be mediated by non-GSK mechanisms in A2B5+ NSC stimulated by 0.5% serum and AICAR. Lithium may affect STAT3 directly or indirectly.

In addition to GSK3β, lithium binds to and inhibits several magnesium-dependent phosphomonoesterases [56], [57] and inositol monophosphatase [58], [59]. Lithium also stimulates phosphoinositol-3-kinase (PI3K) and Akt-1 [60], both of which may negatively regulate STAT3 by reducing its DNA binding activity [61]. Lithium may regulate STAT3 through any of these pathways. Alternatively, lithium may bind and inhibit STAT3 directly.

We hope that our study will direct attention towards lithium's effects on the JAK (Janus kinase) and STAT3 pathway. This pathway not only stimulates astrogliogenesis [30], [48], [51], [62], [63], [64] but also microglial activation [65], [66]. Lithium inhibition of STAT3 would explain the dramatic reduction of activated microglia and macrophage due to lithium treatment of NSC transplanted into spinal cord [21].

STAT3 inhibition may explain lithium's remarkable lack of carcinogenicity. Lithium inhibition of GSK3β increases WNT/beta-catenin, known to be associated with cancer [67], [68], [69]. Yet, millions of people have taken lithium for their lifetime without reports of increased cancer. In fact, lithium reduces formation of some tumors [29], [70], [71], [72], [73]. JAK/STAT3 activation also increases SOCS (suppressors of cytokine signalling), abnormalities of which cause cancer [74], [75]. By inhibiting STAT3, lithium should reduce SOCS levels.

Our finding that lithium inhibits astrogliogenesis at 3 mM should be of interest for those seeking to grow neurons from NSC. At 1 mM, lithium stimulates neurogenesis without inhibiting astrogliogenesis. However, at 3 mM, lithium strongly stimulates neurogenesis and inhibits astrogliogenesis at the same time, without increasing apoptosis. Growing NSC in 3 mM lithium should produce predominantly neuronal cultures while growing them in 1 mM lithium or specific GSK3β blockers will allow astrocytes to grow. To inhibit astrogliogenesis, higher doses of lithium should be used.

Lithium is an attractive therapy for CNS regeneration. It is safe and robustly stimulates proliferation of endogenous [14] and transplanted neural stem cells [21], [40], as well as axonal regeneration [22], [23]. It increases brain concentrations of neurotrophins [14], [76], [77], [78], [79]. We have now shown that lithium suppresses astrogliogenesis by inhibiting STAT3, an effect that other specific GSK3β blockers seem to lack. At 3 mM concentrations, lithium thus may prevent or retard gliosis after brain and spinal cord injury.

In conclusion, lithium stimulates neurogenesis and suppresses astrogliogenesis by NSCs. We hypothesized that lithium blocks STAT3, which induces astrogliogenesis and microglial activation. Lithium, SB216763, and GID5-6 all inhibited GSK3β, prevented inactivation of beta-catenin, and stimulated neurogenesis. However, only lithium blocked STAT3 activation and astrogliogenesis induced by 0.5% serum or the STAT3 agonist AICAR, these findings indicate that lithium blocked STAT3 activation through non-GSK3β mechanisms. Lithium inhibition of STAT3 not only explains why lithium suppresses astrogliogenesis and microglial activation but also may explain the low carcinogenicity of lithium in clinical use.

## Materials and Methods

For the purposes of this article, we use the term “neural stem/progenitor cells” to refer to cells isolated from the subventricular zone of rats. When placed in growth media with epidermal growth factor (EGF) or fibroblast growth factor (FGF), these cells proliferated and produced neural progenitor cells (NPCs) expressing A2B5 and PSA-NCAM, respectively markers for glial-restricted or neuronal-restricted precursors. When placed in neurobasal media with B27 (NB27, Invitrogen), the cells differentiated to express mature neuronal or astroglial markers, respectively Tuj1 and GFAP. We used the following methods to prepare and identify NSC, to sort the cells, to assess proliferation and apoptosis, to quantify GSK3β and STAT3 activation, and to transfect cells with GID5-6 to block GSK3β.

### NSC Preparation and Treatments

We isolated NSCs from neonatal Fischer 344 rats. The Animal Care and Facilities Committee at Rutgers University approved all animal procedures (Protocol: Rat Breeding Colony, NO. 99-032). Newborn rats (P0 or P1) were anesthetized with isoflurane (5%) and decapitated. Under sterile and ice-cold conditions, we removed the brain, dissected out the lateral wall of the lateral ventricle, and dissociated the tissue by gentle trituration with fire-polished Pasteur pipettes [31].

After filtering the tissue suspension with a cell strainer (BD Falcon, San Jose, CA, USA), we plated the cells (2×10^5^ cells per ml) in NSC culture media (DMEM/F12, Gibco, Grand Island, NY, USA) containing B27 (1∶50, Invitrogen, Carlsbad, CA, USA), basic fibroblast growth factor (bFGF, 10 ng/ml, R & D, USA), epidermal growth factor (EGF, 10 ng/ml, R & D, USA), and Penicillin-Streptomycin (Pen-Strep, 100 IU/ml, Invitrogen). We will refer to the growth-factor containing media as NSC growth media.

The cells grew in a 37°C humidified 5% CO_2_ incubator. We added growth factors every day, changed media every 2 days, and passaged the cells after 7 days. We designated first and second passage cells as P1 and P2, and used only P1 or P2 NSCs in this study. After passage, the cells were cultured in plates or cover slips coated with poly-L-lysine (0.01%, Sigma Aldrich, St. Louis, USA) and laminin (10 µg/ml, Invitrogen), placed in NSC culture media for 1–2 days, and then transferred to basic neurobasal medium plus B27 (NB27) for differentiation assays. Lithium chloride (Sigma Aldrich, St. Louis, USA) was dissolved in Milli-Q water, AICAR (Sigma Aldrich, St. Louis, USA) and SB216763 (10 µM, Tocris Bioscience, Ellisville, USA) were dissolved in dimethyl sulfoxide (DMSO, Sigma Aldrich, St.Louis, USA). We added lithium chloride and other drugs to the culture medium after the passage and then assessed the cells by immunocytochemistry or Western blots 2 to 7 days later, depending on the outcome measure used.

### Immunocytochemistry

For immunocytochemistry, we cultured cells on 12-mm poly-L-lysine/laminin coated glass cover slips at a density of 2×10^4^ cells/cover slip. At specified time points, we immersed the cells for 5–20 minutes in 4% phosphate-buffered (0.01 M phosphate) paraformaldehyde, then blocked non-specific binding sites with 10% normal goat serum (Vector) and solubilized lipids with 0.3% Triton-X-100 for 1 hour. For surface antigen A2B5 or PSA-NCAM labeling, the cells were fixed in 4% paraformaldehyde for only 5 minutes and no Triton-X-100 was added to the blocking or washing solution. The fixation, blocking, and solubilization were done at room temperature (RT).

To label cells, we incubated the cells overnight in primary antibodies diluted in phosphate buffered (0.01 M phosphate) solution containing 3% normal goat serum and 0.3% Triton-X-100. Table 1 lists the primary antibodies and their sources. After washing with phosphate-buffered saline (PBS), we applied secondary antibodies corresponding to the primary antibody. The secondary antibodies were conjugated to fluorescent Alexa 568 or 488 (1∶400, Molecular Probes) or rhodamine (TRITC) or FITC-conjugated goat anti-mouse (1∶200, Jackson ImmunoResearch, goat anti-mouse IgM, μ-chain specific). We stained nuclei with Hoechst 33342 (Molecular Probes, 5 µg/ml) and mounted the cover slips with Gelmount.

**Table 1:** Primary Antibodies used for immunocytochemistry.

Antigen	Type	Dilution	Source	Presumptive label
Nestin	Mouse Monoclonal	1∶50	Millipore	Stem cell
GFAP	Rabbit Polyclonal	1∶200	DAKO	Astrocyte
Tuj1	Mouse Monoclonal	1∶1000	Convance	Neuronal
GalC	Mouse Monoclonal	1∶200	Millipore	Oligodendroglia
A2B5	Mouse Monoclonal	1∶200	Sigma	Glial restricted
PSA-NCAM	Mouse Monoclonal	1∶300	Millipore	Neural restricted

Abbreviation:

GFAP (Glial fibrillary acidic protein).

Tuj1 (Neuronal Class III β-Tubulin);

GalC (Galactosylceramidase);

PSA-NCAM (polysialic acid - neural cell adhesion molecule).

We photographed fluorescent images with a Zeiss Axiovert 200 M epifluorescent microscope or a Zeiss LSM510 confocal microscope, using 20× magnification and 0.4 aperture, an AxioCam camera, and Axiovision 4.6 software (Carl Zeiss, Germany). Cell percentage counts were based on numbers of nuclei and situation of the nuclei in cells expressing specific markers. The actual cells number of each lineage was determined by the combination with the percentage counts and the total number of cells in each treatment, as determined by proliferation assay.

### Proliferation and Apoptosis Assays

To assess proliferation, we seeded NPC's on poly-L-lysine/laminin coated 96-well plates with an initial density of 10^4^cells/well. The cells were cultured in NSC medium for 24 hours and then in NB27 medium supplemented with lithium and other drugs. At planned time points, we froze the cells at −70°C overnight, thawed the cells, and quantified DNA using CyQUANT (Invitrogen) Assay and a Fluoroskan Ascent microplate reader (Thermo Labsystem, US.) with excitation wavelength at 485±10 nm and emission detection wavelength at 530±12.5 nm. We verified that the DNA signal relates linearly to cell number according to a standard curve provided by the manufacturer. The data are expressed in mean ± standard error of mean (mean ± sem).

To assess apoptosis, we used terminal deoxynucleotidyl transferase dUTP nick end labeling (DeadEnd™ Fluorometric TUNEL System, Promega). After incubating the cells in NB27 medium supplemented with various treatments for different times, we used 4% paraformaldehyde in PBS to fix the cells for 25 min at RT, permeabilized the cells with 0.1% Triton X-100 and 0.1% sodium citrate for 5 min, washed them several times with PBS, transferred the cells to an equilibration buffer (200 mM potassium cacodylate, 25 mM Tris-HCl, 0.2 mM DTT, 0.25 mg/ml BSA, 2.5 mM cobalt chloride, pH 6.6) for 10 min, and then immersed the slides for 60 min at 37°C in a reaction buffer containing 0.3 U/ml terminal deoxynucleotidyl transferase and 50 µM FITC-fluorescein-12-dUTP. To stop the reaction, we immersed the slides in a stopping solution (300 mM Sodium Chloride, 30 mM sodium citrate, pH 7.0) for 15 min at RT. After staining the nuclei with 4′,6-diamidino-2-phenylindole (DAPI), we mounted the cover slips on slides and visualized the cells with an epifluorescent microscope equipped with standard fluorescein and DAPI filters. We counted TUNEL-positive cells in ≥15 independent fields from each of three experiments and expressed the data as a percent of total DAPI-stained nuclei counted per field.

### Western Blots

To quantify phosphorylated and non-phosphorylated GSK3β and JAK/STAT3 or the other proteins expression, we extracted proteins from the cells using a radio-immunoprecipitation assay buffer (RIPA, Sigma-Aldrich, USA), containing 50 mM Tris-HCl at pH 8.0 with 150 mM NaCl, 1.0% Igepal CA-630 (NP-40), 0.5% sodium deoxycholate, and 0.1% sodium dodecyl sulfate (SDS). After pre-clearing the lysates by centrifuging at 12,000 rpm for 3 minutes, we added phosphatase inhibitor cocktail (1∶50, Sigma) and loaded equal amounts of protein (5–120 µg/sample) on 10% SDS polyacrylamide gel electrophoresis (SDS-PAGE). Rainbow molecular weight marker (GE Healthcare) and a Tris-glycine running buffer (25 mM Tris, 190 mM glycine, 0.1% SDS) were used.

We transferred the gels to PVDF (polyvinylidene difluoride) membranes that had been soaked in methanol for 1 min and equilibrated in transfer buffer containing 25 mM Tris base, 190 mM glycine, 20% methanol, and 0.005% SDS [32]. After blocking with Tris buffered saline (TBS) buffer containing 0.1% Tween (TBST) and 5% non-fat dry milk (TBST+5% milk) for 1 hour at RT, we applied primary antibodies in TBST+5% milk overnight at 4°C, washed with TBST, applied horseradish peroxidase (HRP) conjugated secondary antibody in PBS+5% milk for 1 hour, imaged the bound antibodies using ECL plus Western Blotting Detection System (GE Healthcare, UK), analyzed with Kodak Molecular Imaging Software (v4.4.4), and normalized the phosphorylated protein to non-phosphorylated total protein. Table 2 lists the antibodies used.

**Table 2:** Primary Antibodies used for Western blot.

Antigen	Type	Comments
Phospho-Tyr705-STAT3	Rabbit Monoclonal	Phosphorylated STAT3 at the Tyr705 site.
STAT3	Rabbit Monoclonal	Non-phosphorylated STAT3
Phospho-Beta-catenin	Rabbit Monoclonal	Phosphorylated beta-catenin at Ser33/37/Thr41
Beta-catenin	Rabbit Monoclonal	Non-phosphorylated beta-catenin
Ser9-Phospho-GSK3β	Rabbit Monoclonal	Phosphorylated GSK3β at the Ser9 site
GFAP	Mouse Monoclonal	Maker as mature astrocytes
GAPDH	Rabbit Monoclonal	Housekeeping Gene

Abbreviations:

STAT3 (Signal transducer and activator of transcription 3),

GAPDH (Glyceraldehyde 3-phosphate dehydrogenase.

### Axin GID 5-6 and GID 5-6 LP Plasmid Transfections

Axin is a cytoplasmic protein that binds GSK3β and beta-catenin, allowing the former to phosphorylate the latter [33]. GSK3β Interaction Domain (aa380 to 404, GID5-6) is a 25 amino acid polypeptide fragment of axin that binds GSK3β, inhibiting its activity [34], [35], [36]. Dr. Peter S. Klein provided myc-tagged GID5-6 (Axin GID 380–404/pCS2MT) and GID5-6LP (Axin GID (Leu→Pro) 380–404/pCS2MT). GID5-6LP is an ineffective analog of GID5-6 with proline substituted for leucine.

We used the Amaxa® Rat Neuron 96-well Nucleofector® kit (Lonza VHPG-1006) to transfect 1 µg of GID5-6 or GID5-6LP into NSC suspensions. The Nucleofector® (program 96-EM-110) usually transfects 50–65% of 2.5–3.0×10^5^ cells per well. To identify cells that had been successfully transfected, we immunostained the cells for myc (1∶200, Cat 2278, Cell Signaling Technology) after 24 hours in NSC growth media.

For immunocytochemistry, we grew 3×10^4^cells on cover slips. To assess proliferation, we seeded 10^4^ cells per well and counted before and after 7 days in culture. For protein analyses, we grew the cells in 6-well plates at 5×10^5^cells/well. After transferring to NB27 media, we assessed the cells at 24 hours for GSK3β and STAT3 phosphorylation.

### Statistical Analyses

Data in the figures represent mean±sem and n indicates the number of experiments. We used the Student's unpaired t-test to compare two groups and analysis of variance (ANOVA) to compare multiple groups, followed by Dunnett's posthoc test to compare pairs of groups. A p-value of <0.05 indicates significance.

## Supporting Information

Figure S1 **Lithium but not SB216763 suppresses S100β expression.** NSCs were grown for 7 days in NB27 containing LiCl (0.5, 1.0, 3.0 mM) or SB216763 (10 µM) and then stained for S100β (red); nuclei were stained with Hoechst 33342 (blue). The photomicrographs (A1) show representative fields from each treatment group (Control, 3 mM LiCl and SB21763). The graphs show actual number counts of S100β+ cells (A2), normalized to untreated control counts. Lithium reduced the S100β+ cells number by 0.72±0.08 fold in 0.5 mM LiCl (P<0.05) compared to control, 0.51±0.09 in 1.0 mM LiCl (P<0.05), 0.44±0.11 in 3.0 mM LiCl (P<0.05). In contrast, SB216763 treatment did not reduce astrocyte number (1.1±0.09 fold versus Control, P>0.05). Data are expressed as mean ± sem from three independent experiments (n = 3, * denotes P<0.05 vs. control, one way ANOVA with Dunnett's post-test).
(TIF)

Figure S2 **Both lithium and SB216763 stimulate neurogenesis by NSCs.** NSCs were differentiated in NB27 medium containing LiCl (1.0 mM) or SB216763 (10 µM) for 7–8 days. BrdU (10 µM, Sigma) was added to cultures 2 days before fixation. Cells were stained for Tuj1 (Green) and BrdU (red, A1). The graphs show the percentage of BrdU+ cells in Tuj1+ cells (A2), both LiCl and SB216763 significantly increased the BrdU fraction in Tuj1+ cells. Control group: 4.8±1.2%; lithium (1 mM) group: 14.3±2.8% (P<0.05); SB216763 (10 uM): 25.7±2.6%, (P<0.05). Data are expressed as mean ± sem from three independent experiments (n = 3, * denotes P<0.05 vs. control, one way ANOVA with Dunnett's post-test).
(TIF)

Figure S3 **The co-localization of PSA/NCAM and Tuj1 after 5 days of differentiation.** NSCs were grown for 5 days in NB27 containing LiCl (0.5, 1.0, 3.0, 5 mM) or SB216763 (10 µM) and then stained for Tuj1 (red) and PSA/NCAM (green), nuclei were stained with Hoechst 33342 (blue). Most Tuj1+ cells co-localized with PSA/NCAM after 5 days. The photomicrographs (A1) show representative fields of the co-localization of PSA/NCAM and Tuj1 from each treatment group (control, 1 mM LiCl, SB216763). The graphs show the percentage of Tuj1 and PSA/NCAM double positive cells out of total cells (A2). Data are expressed as mean ± sem from three independent experiments (n = 3, * denotes P<0.05 vs. control, one way ANOVA with Dunnett's post-test).
(TIF)
