# Supplementary figures and images for: Lithium Suppresses Astrogliogenesis by Neural Stem and Progenitor Cells by Inhibiting STAT3 Pathway Independently of Glycogen Synthase Kinase 3 Beta
Source: PLoS One. 2011 Sep 9;6(9):e23341. doi: 10.1371/journal.pone.0023341 (PMC3170293; doi:10.1371/journal.pone.0023341)

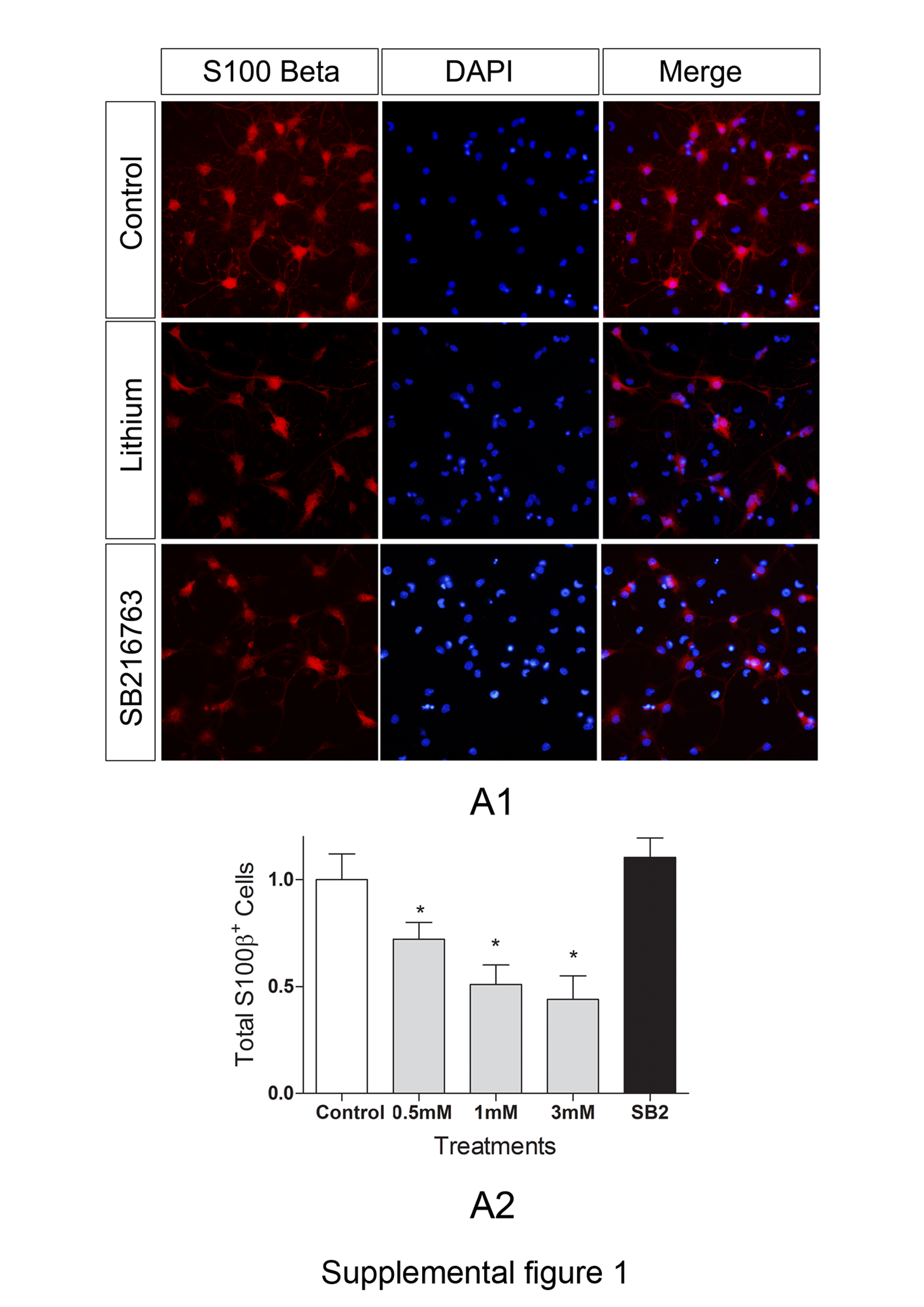

Supplement: Figure S1 — Lithium but not SB216763 suppresses S100β expression. NSCs were grown for 7 days in NB27 containing LiCl (0.5, 1.0, 3.0 mM) or SB216763 (10 µM) and then stained for S100β (red); nuclei were stained with Hoechst 33342 (blue). The photomicrographs (A1) show representative fields from each treatment group (Control, 3 mM LiCl and SB21763). The graphs show actual number counts of S100β+ cells (A2), normalized to untreated control counts. Lithium reduced the S100β+ cells number by 0.72±0.08 fold in 0.5 mM LiCl (P<0.05) compared to control, 0.51±0.09 in 1.0 mM LiCl (P<0.05), 0.44±0.11 in 3.0 mM LiCl (P<0.05). In contrast, SB216763 treatment did not reduce astrocyte number (1.1±0.09 fold versus Control, P>0.05). Data are expressed as mean ± sem from three independent experiments (n = 3, * denotes P<0.05 vs. control, one way ANOVA with Dunnett's post-test). (TIF) [file pone.0023341.s001.tif]

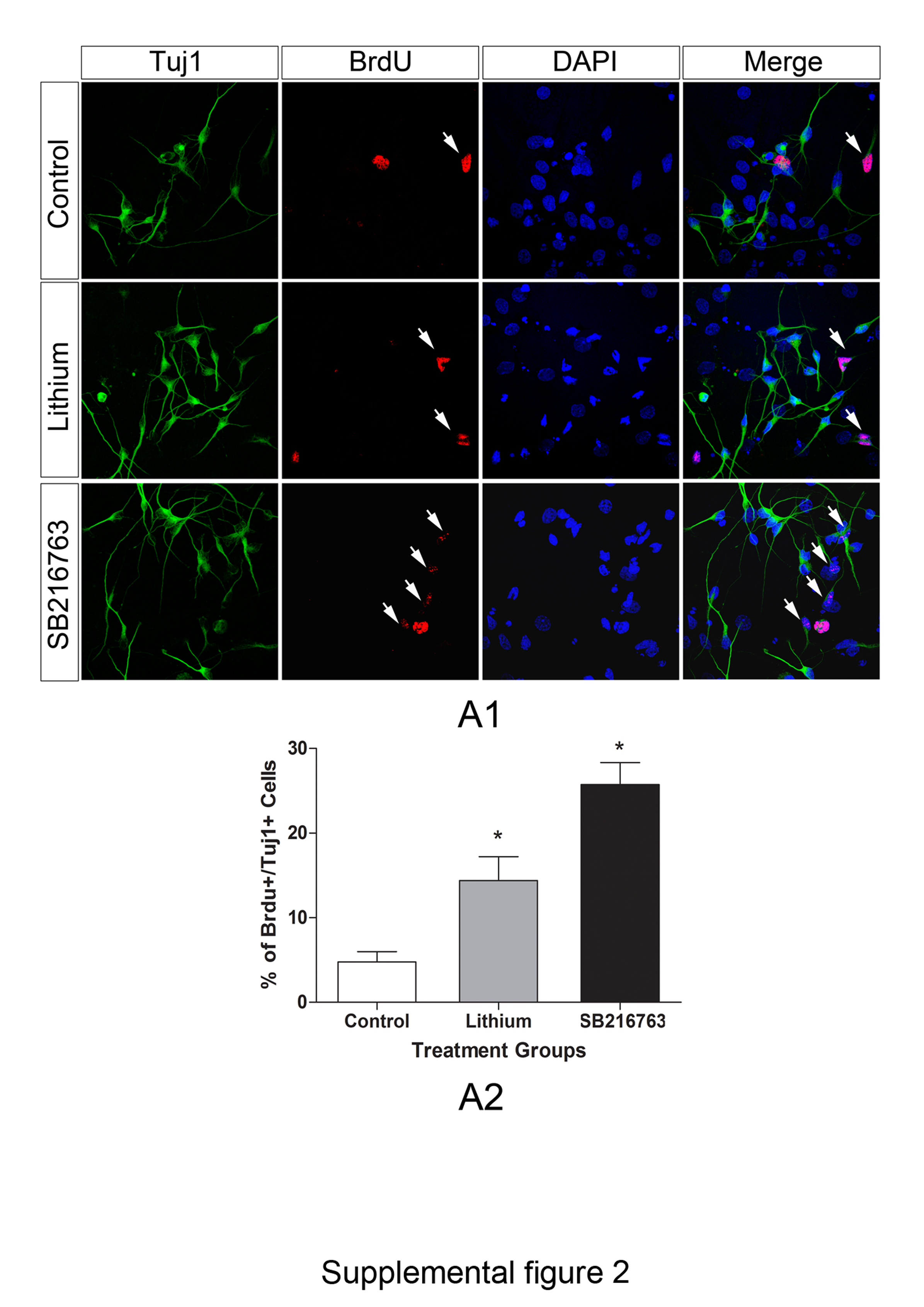

Supplement: Figure S2 — Both lithium and SB216763 stimulate neurogenesis by NSCs. NSCs were differentiated in NB27 medium containing LiCl (1.0 mM) or SB216763 (10 µM) for 7–8 days. BrdU (10 µM, Sigma) was added to cultures 2 days before fixation. Cells were stained for Tuj1 (Green) and BrdU (red, A1). The graphs show the percentage of BrdU+ cells in Tuj1+ cells (A2), both LiCl and SB216763 significantly increased the BrdU fraction in Tuj1+ cells. Control group: 4.8±1.2%; lithium (1 mM) group: 14.3±2.8% (P<0.05); SB216763 (10 uM): 25.7±2.6%, (P<0.05). Data are expressed as mean ± sem from three independent experiments (n = 3, * denotes P<0.05 vs. control, one way ANOVA with Dunnett's post-test). (TIF) [file pone.0023341.s002.tif]

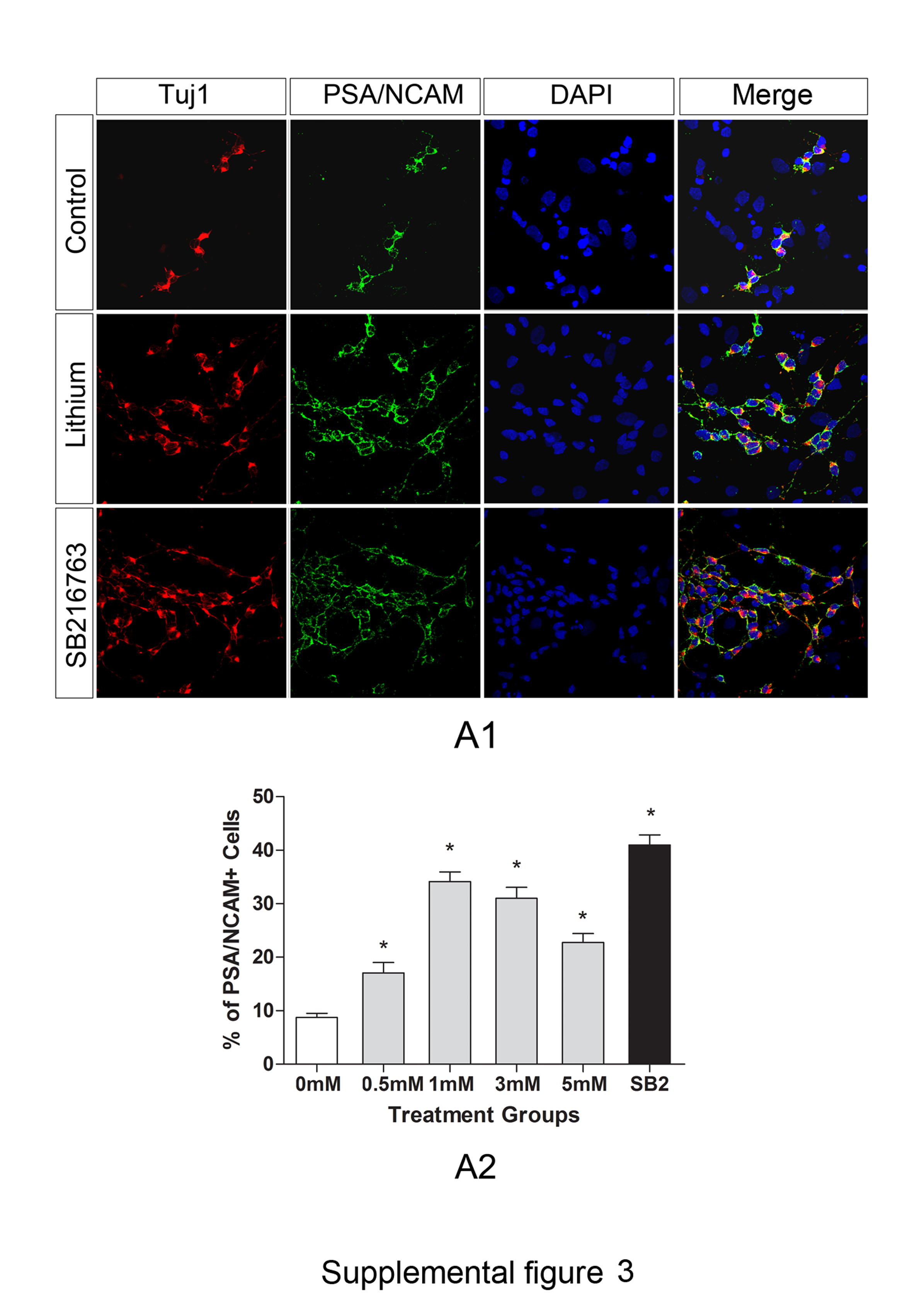

Supplement: Figure S3 — The co-localization of PSA/NCAM and Tuj1 after 5 days of differentiation. NSCs were grown for 5 days in NB27 containing LiCl (0.5, 1.0, 3.0, 5 mM) or SB216763 (10 µM) and then stained for Tuj1 (red) and PSA/NCAM (green), nuclei were stained with Hoechst 33342 (blue). Most Tuj1+ cells co-localized with PSA/NCAM after 5 days. The photomicrographs (A1) show representative fields of the co-localization of PSA/NCAM and Tuj1 from each treatment group (control, 1 mM LiCl, SB216763). The graphs show the percentage of Tuj1 and PSA/NCAM double positive cells out of total cells (A2). Data are expressed as mean ± sem from three independent experiments (n = 3, * denotes P<0.05 vs. control, one way ANOVA with Dunnett's post-test). (TIF) [file pone.0023341.s003.tif]
